# Penetrating Heart Injury due to Screwdriver Assault

**DOI:** 10.1155/2015/140507

**Published:** 2015-04-05

**Authors:** P. A. Dieng, M. S. Diop, A. G. Ciss, P. S. Ba, S. Diatta, M. Gaye, M. L. Fall, A. Ndiaye, M. Ndiaye

**Affiliations:** Service de Chirurgie Cardiovasculaire et Thoracique, CHUN Fann, Dakar, Senegal

## Abstract

Penetrating heart injuries cause wounds in the cardiac chambers. Most of them are due to gunshot or stabbing by knives. Screwdriver is an uncommon weapon. Authors report a case of stab wound by screwdriver, treated at cardiovascular center in Dakar. This is a 16-year-old boy who experienced physical aggression. He was assaulted with a screwdriver and had stab wound on the anterior wall of the chest. Physical examination showed a screwdriver penetrating the sternum bone over a right angle. He had a mild pericardial blood effusion and a right ventricle wound 5 mm in diameter with transection of the right coronary vein. The screwdriver was removed without cardiopulmonary bypass (CPB) and the ventricle wound repaired by direct suture of stitches reinforced with Teflon pledgets. The right coronary artery was ligated. Postoperative period was free of events. Screwdriver is uncommonly used as a weapon. It is a dangerous device because of its rigid structure and narrow tip.

## 1. Introduction

Penetrating heart injuries cause wounds in cardiac chambers. Most of them are due to gunshot or stabbing by knives. Screwdriver is an uncommon weapon which induces stab wound in frontal injury of the chest.

After penetrating heart injury the majority of patients die before getting to the hospital. In medical facilities, the 2 most common clinical presentations of cardiac wounds are pericardial tamponade and excessive hemorrhage [1] with shock. The surgical care should be done urgently; however the outcomes depend on the accurate indication and physical lesions.

Authors report a case of stab wound by screwdriver, treated at cardiovascular center in Dakar.

## 2. Case Presentation

This is a 16-year-old boy who experienced physical aggression in urban fight. He was assaulted by a screwdriver and had stab wound on the anterior face of the chest. He was transported from St. Louis, 192 km away, to our facility by ambulance in a stable hemodynamic status and arrived 8 hours later.

**Figure 1:**
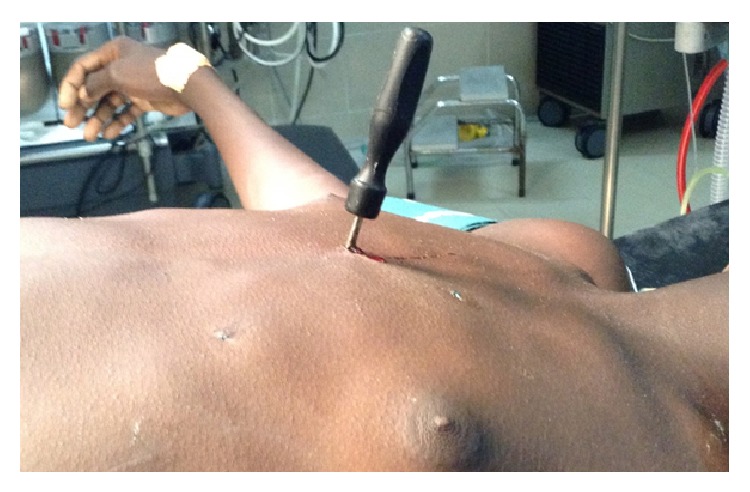
Screwdriver penetrating the chest.

Physical examination showed a screwdriver penetrating the sternum bone in the inferior third over a right angle (Figure 1). Heart bruits were normal. Signs of important bleeding were not seen.

Cardiac ultrasound showed a metallic foreign body in the right ventricle wall with images of thrombus in the right ventricle and a mild pericardial effusion.

**Figure 2:**
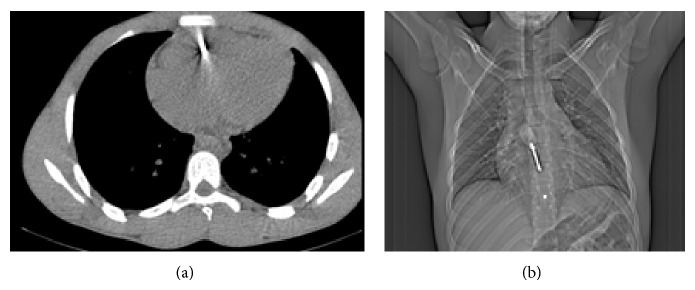
CT scan images of screwdriver inside the heart.

The CT scan showed the screwdriver landing into the right ventricle (Figure 2).

**Figure 3:**
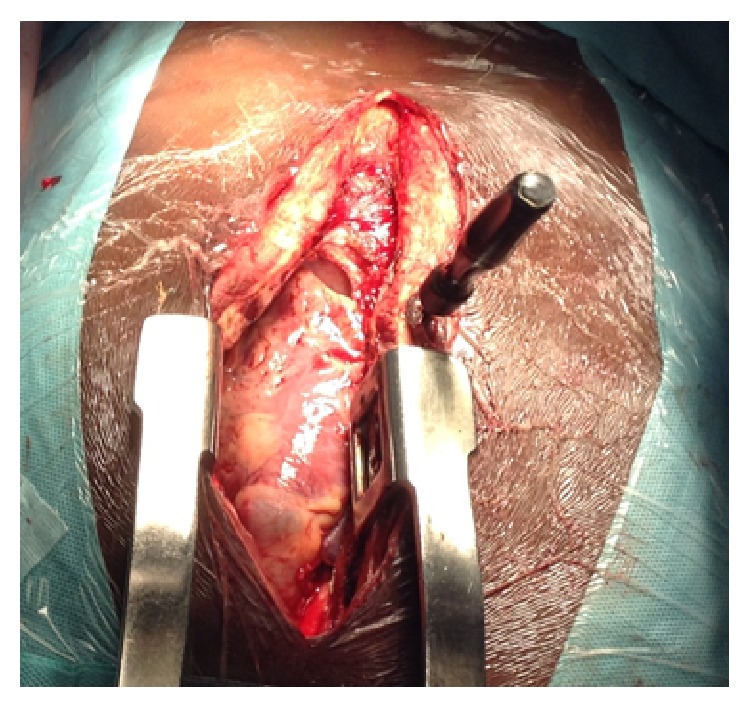
Image of screwdriver remaining after sternotomy.

Surgical exploration was done under general anesthesia and orotracheal intubation. Surgical access was a median sternotomy (Figure 3).

**Figure 4:**
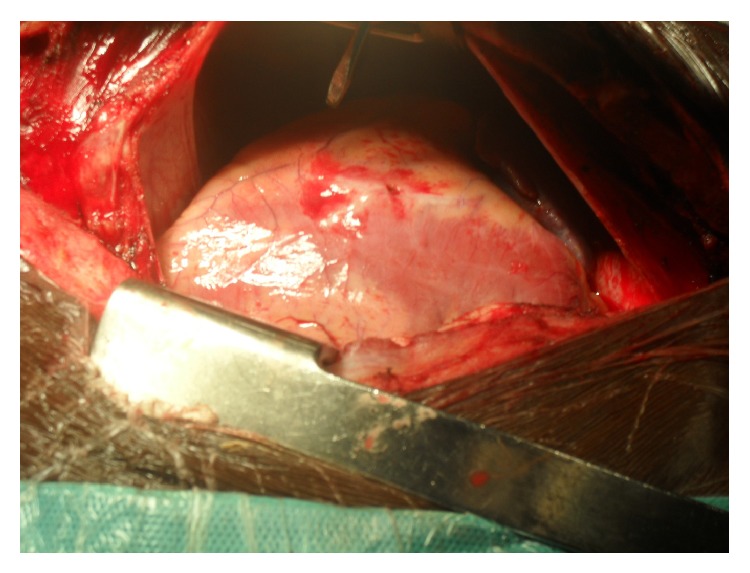
Image of heart stab wound with vein injury after screwdriver removal.

We discovered a mild pericardial blood effusion and a right ventricle wound of 5 mm in diameter with transection of the right coronary vein (Figure 4).

The screwdriver was removed without cardiopulmonary bypass (CPB) and the ventricle wound repaired by direct suture of stitches reinforced with Teflon pledgets. The right coronary vein was ligated.

Postoperative period was free of events. No clinical or biological sign of infection was noted.

Cardiac ultrasound done the day after surgery showed a small thrombus in the lateral wall of the right ventricle. Under heparin therapy, that thrombus disappeared on the 7th day of follow-up.

## 3. Discussion

Penetrating heart injuries are extremely urgent. Only 11 to 25% of patients arrive to hospital with signs of life [1, 2]. Among those patients, 20% have stable hemodynamic status like our patient. The cardiac wound was sealed off by the weapon itself, the screwdriver which remains impacted into the sternum and into the heart chamber. This positive situation permits a surgical care with good results and survival of patient. The survival rate is 89% in the literature [3]. However stab wounds are less lethal than gunshot wounds. Cardiac tamponade or major bleeding leads to unstable hemodynamic status. Preoperative and operative resuscitation are essential for life salvage.

Usually cardiac ultrasound is enough for diagnosis [4], but in patient with stable blood pressure such as this case, CT scan gives more information about cardiac wound and presence of pericardial blood effusion [5].

For surgical access, median sternotomy is widely used even though thoracotomy can be used as well [6]. The sternotomy allows better view of frontal injuries and permits the repair of the majority of lesions.

The most frequently injured chamber is the right ventricle in cardiac wound [7] as it is in our case.

Cardiac cavities repair is mostly done without cardiopulmonary bypass (CPB) [8]. Nowadays CPB is recommended for repair of severe or multiple lesions. Coronary artery transection is uncommon [7, 8] but occured sometime and could be lethal. In our case the coronary lesion was located in the right vein and its ligation in such young patient was accurate.

The penetrating cardiac wounds are mostly due to bullet or stabbing [1, 9]. Gunshot wounds are more likely to result in death than stabbing wounds of the heart [10]. An isolated cardiac stab wound is a relatively innocent injury in a patient at a hospital accustomed to managing penetrating trauma [11].

Penetrating heart traumas were generally observed in young patients with low socioeconomic status [12]. Screwdriver is uncommonly used as a weapon. It is a dangerous device because of its rigid structure and narrow tip. That appearance allows the fracture of the sternum and a stab wound of the heart. Despite the long time that foreign body remained in the sternum (for several hours in this case) we do not have sternum osteomyelitis or endocarditis, as described in some cases [5, 6].

## 4. Conclusion

Heart injury by screwdriver assault is a rare situation. Leaving that foreign body impacted into the chest until emergency room care is important for life salvage.
